# Supplementary material for: Optimization of planting dates of Jalapeno pepper (Capsicum annuum 'Jalapeño' L.) and cantaloupe (Cucumis melo var. cantalupensis Ser.) relay cropped with strawberry (Fragaria × ananassa Duchesne)
Source: PLoS One. 2020 Jul 27;15(7):e0236677. doi: 10.1371/journal.pone.0236677 (PMC7384653; doi:10.1371/journal.pone.0236677)
Supplement: Attachment — Submitted filename: Response to reveiwers.docx [file pone.0236677.s003.docx]

Dear Editor,

Plos One

Please find attached our manuscript titled ‘Optimization of planting dates of Jalapeno pepper (*Capsicum annuum* 'Jalapeño' L.) and cantaloupe (*Cucumis melo* var. *cantalupensis* Ser.) relay cropped with strawberry (*Fragaria × ananassa* Duchesne)’. The authors appreciate reviewers’ comments and suggestions made on the manuscript. In the revised document, the authors have addressed all the comments and added the necessary information as requested. All the changes are track marked and highlighted in the attached documents with line numbers.

Best,

Ravneet K Sandhu

**Editors comments:**

| I suggest the authors to include in this part of the manuscript data about climate and, in particular, to report mean air temperature and rainfall values of the test area with reference to previous 5/10 years, please. This is fundamental to understand the environmental characteristics of the test area. | Table on temperature and rainfall data recorded in Balm, Florida is now included. |
| --- | --- |
| In the Materials and Methods, the authors do not report any information on irrigation and fertilization rates of the main species in the study. Please, provide. | Authors have provided the information indicated in comments. |
| In my opinion, discussion should be improved. | Discussion is revised |
| Conclusions are supported by the data but tend to repeat part of the results. In my opinion, the conclusions should be improved, and authors should pay more attention on the impact of their results agriculture. | Conclusions are revised |
| I suggest the authors to improve the quality of tables and figures in agreement with the Guide for Authors of PLOS ONE. | The figures were revised with help of diagnostic tool ( [https://pacev2.apexcovantage.com/](https://urldefense.proofpoint.com/v2/url?u=https-3A__pacev2.apexcovantage.com_&d=DwMGaQ&c=sJ6xIWYx-zLMB3EPkvcnVg&r=j3QITV2cYLKQU-K_uDbUmg&m=cxWpr8cQ6K3LVgM9q0r04Nw4ywwRrJqFdTUNP5DOGIQ&s=iiiMQWLEt-QxgfUUVZvLBPJr_q0njJmHET9C1WZWO9Q&e=)) suggested. |
| And also the reference section should be re-checked. | Reference section is revised |

| **Reviewer comments** | **New line number and remarks** |
| --- | --- |
| However, the authors did not observe in a deep way the yield traits of the cultivated species such as, total production, marketable production, number of fruit and average fruit weight. | Only the marketable fruits were picked, and number of fruit data is included. Fruit yield included both weight and fruit number. |
| Furthermore, the authors did not look on the influence of the treatments on fruit nutritional and functional properties of the vegetables and strawberry | The study was mainly focused on grower’s aspect (growth/development and yield). The nutritional status part is not under the scope of study. However, the authors still included some discussion on the comment indicated. |
| Line 55: Instead of: ...Comopetition...it should be: .... Competition... | Line 56: the typo was corrected |
| Line 61: missing black | Line 62: included blank |
| Line 79: Instead of: …21-26 C…it should be: …21-26 °C | Line 81: a degree symbol is inserted |
| Information on the effect of cultivation practices on yield and yield related traits, nutritional and functional properties of the involved vegetables and strawberry should be provided. | Comment is addressed above |
| Since the experiments were repeated twice, in the ANOVA analysis, please include the year as fix factor. | Year was considered as a fixed factor in initial analysis, that’s why two-year data is presented separately. It is also mentioned in data analysis section. |
| Please add, also, a heat map (please see the following link [https://biit.cs.ut.ee/clustvis/](https://urldefense.proofpoint.com/v2/url?u=https-3A__biit.cs.ut.ee_clustvis_&d=DwQGaQ&c=sJ6xIWYx-zLMB3EPkvcnVg&r=j3QITV2cYLKQU-K_uDbUmg&m=cxWpr8cQ6K3LVgM9q0r04Nw4ywwRrJqFdTUNP5DOGIQ&s=C-2Xg4EUiXS9yJYblTP1OID90Q-0irrk8oriFqfkLZ8&e=)), which might provide an extra value to the work. | Comment is addressed as indicated by the editors |
| Please re-write the paragraph accordingly to the revised statistical analysis (ANOVA analysis and heat map). | Comment addressed |
| Please, try to explain in a deeper way the results. I miss some speculations concerning some physiological processes involved… | Comment addressed |
| Conclusions - Line 366: missing black | Conclusion section is revised |
| - Line 370: Instead of … alone Unlike…it should be… alone. Unlike | Conclusion section is revised |
| Line 371: Instead of … without yiesld loss…it should be… without yield loss… | Conclusion section is revised |
